# Prognostic Value of Inflammatory Biomarkers in Patients With Stage I Lung Adenocarcinoma Treated With Surgical Dissection

**DOI:** 10.3389/fonc.2021.711206

**Published:** 2021-09-01

**Authors:** Yu-Jia Shen, Li-Qiang Qian, Zheng-Ping Ding, Qing-Quan Luo, Heng Zhao, Wu-Yan Xia, Yuan-Yuan Fu, Wen Feng, Qin Zhang, Wen Yu, Xu-Wei Cai, Xiao-Long Fu

**Affiliations:** ^1^Department of Radiation Oncology, Shanghai Chest Hospital, Shanghai Jiao Tong University, Shanghai, China; ^2^Shanghai Jiao Tong University School of Medicine, Shanghai, China; ^3^Department of Thoracic Surgery, Shanghai Lung Cancer Center, Shanghai Chest Hospital, Shanghai Jiao Tong University, Shanghai, China

**Keywords:** lung adenocarcinoma, neutrophil-to-lymphocyte ratio, systemic inflammation response index, systemic immune-inflammation index, nomogram

## Abstract

**Objective:**

Inflammation plays a crucial role in tumorigenesis and progression. Our purpose was to investigate the prognostic value of neutrophil-to-lymphocyte ratio (NLR), systemic inflammation response index (SIRI) and systemic immune-inflammation index (SII), and develop a nomogram to predict the cancer-specific survival (CSS) and disease-free survival (DFS) of stage I lung adenocarcinoma patients.

**Methods:**

1431 patients undergoing surgical resection with pathologically confirmed stage I lung adenocarcinoma were reviewed. The optimal cut-off values for NLR, SII, and SIRI were defined by the receiver operating characteristic (ROC) curve. Cox proportional hazards regression analyses were performed to recognize factors significantly correlated with CSS and DFS to construct the nomogram. The value of adjuvant chemotherapy on model-defined high-risk and low-risk patients was further explored.

**Results:**

The cohort had a median follow-up time of 63 months. Multivariate analysis revealed that higher NLR (≥2.606), higher SIRI (≥0.705), higher SII (≥580.671), later T stage, histological pattern with solid or micropapillary components and radiologic features with solid nodules were significantly associated with worse CSS and DFS. The concordance index (C-index) of the nomogram established by all these factors was higher than that of the TNM staging system both in CSS (validation set 0.778 vs 0.652) and DFS (validation set 0.758 vs 0.695). Furthermore, the value of the established nomogram on risk stratification in stage I lung adenocarcinoma patients was validated.

**Conclusions:**

Higher NLR, SII and SIRI pretreatment were associated with worse survival outcomes. A practical nomogram based on these three inflammatory biomarkers may help clinicians to precisely stratify stage I lung adenocarcinoma patients into high- and low-risk and implement individualized treatment.

## Introduction

Lung cancer is the first leading cause of cancer deaths worldwide (1), with lung adenocarcinoma accounting for the majority. Only 21% of patients are diagnosed at stage I. Despite the early stage, the 5-year survival rate is about 73%-90% (2, 3). Surgical resection remains the standard of care for stage I lung adenocarcinoma. The cumulative incidence of 5-year local-recurrence and distant-metastasis is approximately 12%-15% and 17%-22% (4, 5). Stage IB patients with high-risk factors may be considered with adjuvant chemotherapy on the basis of National Comprehensive Cancer Network (NCCN) guidelines (6). How to select patients with a high risk of recurrence and metastasis is one of the academic research hotspots. Many prognostic factors have been summarized to recognize high-risk individuals, such as tumor size, wedge resection, differentiation grade, and visceral pleural involvement (7). Nevertheless, patients’ immune and inflammatory indices are not included, which may be the same critical.

It is known that inflammation plays an indispensable role in cancer development, tumor angiogenesis and metastasis (8–10). The local immune response induced by tissue injury as well as systemic inflammation mediates the initiation and progression of carcinogenesis (11). NLR, as a representative parameter of systemic inflammation, is considered to be valuable in prognosis (12). Many studies revealed that high NLR could serve as an indicator of poor prognosis in patients with many solid tumors, such as pancreatic cancer, gastric cancer, cervical cancer, liver cancer, hepatocellular carcinoma, colorectal cancer, and non-small cell lung cancer (NSCLC) (13–18). Recently, SII and SIRI were developed and seemed to have a stronger prognostic power (19, 20). However, few previous studies to date focused on patients with stage I lung adenocarcinoma in such a big sample size. Multi histological types and TNM stages may lead to selection bias and heterogeneity. Furthermore, as far as we know, no prediction model includes inflammation markers for stage I adenocarcinoma.

This study retrospectively analyzed the prognostic significance of NLR, SII and SIRI in surgically resected stage I lung adenocarcinoma patients and established a prognostic nomogram incorporating inflammation-based biomarkers.

## Materials and Methods

### Patients

Patients diagnosed with stage I lung adenocarcinoma pathologically after receiving complete surgical resection at Shanghai Chest Hospital between January 2014 and December 2015 were retrospectively reviewed. The inclusion criteria included: pathologically confirmed primary stage I (T1/T2aN0M0) lung adenocarcinoma according to the criteria of the American Joint Committee on Cancer (AJCC 8^th^); a history of complete surgical resection (lobectomy, pneumonectomy); documentation of complete blood counts (CBCs) within 1 week before the operation. Patients were excluded from undergoing sub-lobar resection (segmentectomy, wedge resection) or no lymph node dissection; R1 or R2 resection; treated with neoadjuvant chemotherapy or chemoradiotherapy; a history of the hematological disease, autoimmune disease and infection; no information on extracted data.

### Data Collection

Patients’ clinicopathologic characteristics and routine blood results were obtained from medical records. The clinicopathologic variables such as age, gender, smoking history, tumor location, tumor size, histological classification and radiologic features were collected. Subsolid nodules were defined as nodules with ground-glass opacity (GGO) components in radiologic evaluation. The NLR, SII, and SIRI were calculated as follows: NLR= neutrophil count/lymphocyte count; SII= neutrophil count × platelet count/lymphocyte count; SIRI= neutrophil count × monocyte count/lymphocyte count.

Follow-up information was obtained retrospectively through electronic medical records and telephone interviews. After surgery, the patients underwent routine follow-up once every 2-3 months for the first 2 years, every 6 months for the next 2 years, and annually thereafter. The last follow-up date was December 16, 2020.

### Statistical Analysis

The outcomes of this study were CSS and DFS. CSS was defined as the time from the date of surgery to death due to lung cancer or the last follow-up. DFS was defined as the time between the day of surgery to the earliest recurrence, lung cancer-related death, or last follow-up. Categorical variables were presented as numbers and percentages, whereas continuous variables were showed by the median and range. Chi-square was used to compare differences between categorical variables and t-test was performed to investigate comparisons of numerical variables.

The ROC curve analysis was performed to identify the optimal cut-off values for NLR, SII and SIRI by calculating the maximum Youden index. A logistic regression model was performed to find independent clinicopathologic variables associated with NLR, SII and SIRI. Variables associated with each inflammatory biomarker in the univariate logistic regression were incorporated in the multivariate logistic regression analyses. CSS and DFS rates were estimated with the Kaplan-Meier survival method and compared by the log-rank test. Cox regression model was performed to detect independent predictors of CSS and DFS. The significant variables detected by univariate survival analysis were then subjected to the multivariate regression model.

Thereafter, all patients we included in this study were classified into the nomogram training cohort and validation cohort in a proportion of 7:3 randomly. We established a prognostic nomogram to predict CSS and DFS in the training set based on the outcomes of the multivariate Cox regression model. The C-index was calculated to determine the discrimination ability both in the training and validation cohort. ROC curve was performed to compare the sensitivity and specificity of survival prediction. Furthermore, bootstrap resampling with 1000 times was performed to prevent the overestimation of the established nomogram and calibrate the evaluation.

Finally, to investigate the impact of adjuvant chemotherapy on patients divided by the nomogram, stage IB patients were included. Because of the imbalanced data between observation and adjuvant chemotherapy groups, propensity score matching (PSM) analysis was conducted to correct for selection bias. PSM accounted for variables including age, gender, smoking history, tumor location, radiologic features, histological classification, and inflammation-based biomarkers status. Patients were matched using one-to-one nearest-neighbor matching without replacement. The caliper used for matching was set at 0.01.

Variables with all two-sided P<0.05 were considered statistically significant. All statistical analyses were accomplished by SPSS statistical software (version 26) and R software (version 3.6.2).

## Results

### Patient Characteristics

Records for a total of 1868 Patients with stage I lung adenocarcinoma who received surgical resection were identified for this study. After examining the medical records, 403 were excluded from receiving sub-lobar resection, one was excluded from R1 or R2 resection, 5 were excluded from receiving neoadjuvant chemotherapy. Of the remaining 1459 patients, 1431 patients had documentation of CBCs within 1 week before surgery. Finally, a total of 1431 patients pathologically diagnosed as resected lung adenocarcinoma were finally included **(****Supplementary Figure 1****)**. Patients’ baseline characteristics were summarized in **Table 1**.

**Table 1:** Clinicopathological characteristics of the patients.

Patient characteristics (n=1431)	Median (range or %)
**Age, years**	60.3 (25-82)
**Gender**	
Male	607 (42.4%)
Female	824 (57.6%)
**Family history**	
Yes	63 (4.4%)
No	1368 (95.6%)
**Smoking history**	
Yes	259 (18.1%)
No	1172 (81.9%)
**Location**	
Right	901 (63.0%)
Left	530 (37.0%)
**Lung lobe**	
Upper lobe	810 (56.6%)
Middle lobe	123 (8.6%)
Lower lobe	498 (34.8%)
**T stage**	
T1	1061 (74.1%)
T2a	370 (25.9%)
**Histological classification**	
Acinar predominant	584 (40.8%)
Papillary predominant	487 (34.0%)
Solid predominant	76 (5.3%)
Lepidic predominant	207 (14.5%)
Micropapillary predominant	13 (0.9%)
others	64 (4.5%)
**Histological pattern**	
SMPP	89 (6.2%)
SMPM	374 (26.1%)
SMPN	968 (67.6%)
**Lymphovascular invasion**	
Yes	22 (1.5%)
No	1409 (98.5%)
**Radiologic features**	
Solid	854 (59.7%)
Subsolid	577 (40.3%)

SMPP, solid/micropapillary-predominant; SMPM, solid/micropapillary-minor; SMPN, solid/micropapillary-negative.

There were 607 males (42.4%) and 824 females (57.6%), with a median age of 60.2 years (range, 25-82y). According to the TNM 8th edition, 1061 (74.1%) patients were diagnosed as stage IA and 370 (25.9%) patients were stage IB. In terms of the predominant pattern of the histological classification, there were 584 (40.8%) with acinar predominant, 487 (34.0%) with papillary predominant, 76 (5.3%) with solid predominant, 207 (14.5%) with lepidic predominant, 13 (0.9%) with micropapillary predominant, and 64(4.5%) others which mostly were invasive mucinous adenocarcinoma. To reach a better understanding, patients were divided into three groups according to the proportion of solid or micropapillary components. Solid/micropapillary-predominant (SMPP) was defined as patients with a predominant subtype of solid/micropapillary components. Solid/micropapillary-minor (SMPM) was defined as patients with a solid/micropapillary pattern but not the predominant one, and those without these two components were classified as solid/micropapillary-negative (SMPN). The 1431 patients included 89 (6.2%) in the SMPP group, 374 (26.1%) in the SMPM group, and 968 (67.6%) in the SMPN group.

The median follow-up time was 63.0 months (range, 1.0–82.0 months). The median CSS has not reached, with the 5-year CSS rate was 93.9%.

### Optimal Cut-Off Value for Biomarkers (NLR, SII, SIRI)

Using CSS as the endpoint, we performed ROC curves to determine the optimal cut-off values of these three biomarkers. Based on the maximum Youden index, the optimal cut-off values for NLR, SII, SIRI were 2.606, 580.671 and 0.705, respectively **(****Supplementary Figure 2****)**. The area under the curves (AUCs) of NLR, SII, SIRI were 0.638 (95% CI: 0.571–0.705, P<0.001), 0.627 (95% CI: 0.561–0.693, P<0.001) and 0.659 (95% CI: 0.597–0.721, P<0.001), respectively. Consequently, patients were classified into two groups based on the optimal cut-off values of pretreatment NLR, SII and SIRI in the further survival analysis.

### Correlation Between the Clinicopathologic Variables and Inflammation-Based Biomarkers

The association between NLR and clinicopathologic indices of patients with stage I lung adenocarcinoma was listed in **Table 2**. Gender (P=0.004), smoking history (P=0.001) and T stage (P=0.002) were significantly associated with pretreatment NLR according to univariate logistic regression. Stepwise multivariate logistic regression identified only 2 variables significantly associated with pretreatment NLR: smoking history (P=0.040) and T stage (P=0.003). The patients with higher NLR (≥ 2.606) were more likely with a smoking history and higher pathological T stage. Similar to NLR, patients with higher SII (≥ 580.671) were more likely with a smoking history (**Table S1****)**. Nevertheless, patients with higher SIRI (≥ 0.705) were more likely to be male and relatively older (**Table S2****)**.

**Table 2:** Univariate and multivariate logistic regression associating clinicopathologic variables with NLR.

Characteristics	Univariate analysis			Multivariate analysis		
	OR	95%CI	P-value	OR	95%CI	P-value
**Age, years**			0.098			
<60	Ref.					
60-70	1.195	0.885-1.612	0.245			
≧̸70	1.522	1.034-2.240	0.033			
**Gender**						
Male	Ref.					
Female	0.669	0.510-0.878	0.004*	0.833	0.597-1.161	0.280
**Smoking history**						
No	Ref.					
Yes	1.732	1.256-2.387	0.001*	1.508	1.020-2.229	0.040*
**Location**						
Left	Ref.					
Right	0.762	0.578-1.003	0.053			
**T stage**						
T1	Ref.					
T2a	1.598	1.194-2.140	0.002*	1.556	1.159-2.088	0.003*
**Histological pattern**			0.158			
SMPN	Ref.					
SMPM	1.137	0.834-1.550	0.416			
SMPP	1.622	0.974-2.701	0.063			
**LVI**						
No	Ref.					
Yes	0.725	0.213-2.468	0.607			
**Radiologic features**						
Solid	Ref.					
Subsolid	1.101	0.834-1.454	0.497			

LVI, lymphovascular invasion; NLR, neutrophil-to-lymphocyte ratio; SMPP, solid/micropapillary-predominant; SMPM, solid/micropapillary-minor; SMPN, solid/micropapillary-negative; CI, confidence interval; Ref, reference; OR, odds ratio; *Statistically significant (P < 0.05).

### Survival Analysis

**Figure 1:**
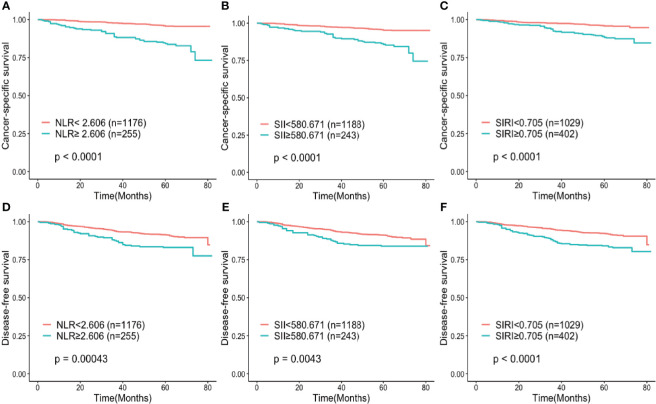
Kaplan–Meier curves for **(A–C)** cancer-specific survival and **(D–F)** disease-free survival according to NLR, SIRI and SII. **(A, D)** Patients were stratified by NLR group; **(B, E)** Patients were stratified by SII group; **(C, F)** Patients were stratified by SIRI group. NLR, neutrophil-to-lymphocyte ratio; SII, systemic immune-inflammation index; SIRI, systemic inflammation response index.

After a median follow-up of 63 months, 99 patients (6.9%) died and 89 deaths (6.2%) were related to lung cancer. Among the other 10 patients, one died of pulmonary embolism, 2 of other tumors, 3 of cerebral hemorrhage and 4 of accidents. The 3-year and 5-year CSS rates for all patients were 96.3% and 93.9%, respectively. In terms of inflammatory markers, the 5-year CSS rates were 84.4% and 95.8% for patients in the high NLR and low NLR group, 88.5% and 95.9% for patients in the high and low SIRI group, 86% and 95.4% for patients in the high and low SII group, respectively. The 3-year and 5-year DFS rates for all included patients were 93.3% and 90.1%, respectively. Survival curves, estimated by the Kaplan-Meier method, indicated that the patients in the high NLR, SII and SIRI groups had both a poorer CSS (P < 0.001) and DFS (P < 0.001) than those in the low NLR, SII and SIRI groups **(****Figure 1****)**.

Factors that impact CSS and DFS identified by univariate and multivariate analysis were summarized in **Tables 3**, **4**. Since solid and micropapillary components seemed to be more clinically significant, histological patterns (SMPN/SMPM/SMPP) were incorporated into the Cox regression model. In the univariate analysis, gender, T stage, histological patterns, radiologic features, NLR, SII, SIRI were all significant prognostic indicators of CSS and DFS, with age and lymphovascular invasion also valuable in predicting CSS and DFS, respectively. Because of the strong collinearity of NLR, SII and SIRI, we put them separately into the multivariate analysis. In the three multivariable modes, higher NLR (CSS: HR 3.756, 95% CI: 2.446-5.766, P<0.001; DFS: HR 1.666, 95% CI:1.150-2.413, P=0.007), higher SII (CSS: HR 3.545, 95% CI: 2.306-5.449, P<0.001; DFS: HR 1.679, 95% CI: 1.146-2.460, P=0.008) and higher SIRI (CSS: HR 2.281, 95% CI: 1.477-3.524, P<0.001; DFS: HR 1.781, 95% CI: 1.267-2.504, P=0.001) were all identified as independent prognostic factors for worse CSS and DFS in invasive stage I lung adenocarcinoma. Also, later T stage, histological pattern with solid or micropapillary components and radiologic features with solid nodules were significantly associated with poorer CSS and DFS (P<0.05).

**Table 3:** Univariate and multivariate cox regression analysis of factors associated with cancer-specific survival.

Characteristics	Univariate analysis			Multivariate (model 1 NLR)				Multivariate (model 2 SII)			Multivariate (model 3 SIRI)		
	HR	95%CI	P	HR	95%CI		P	HR	95%CI	P	HR	95%CI	P
												
**Gender:** Male/Female	0.447	0.292-0.685	<0.001	0.697		0.448-1.084	0.109	0.657	0.422-1.021	0.062	0.738	0.470-1.159	0.738
**Age**			0.070							0.039			0.145
<60y	Ref			Ref				Ref			Ref		
60-70y	1.501	0.933-2.414	0.094	1.486		0.920-2.401	0.105	1.555	0.959-2.521	0.073	1.412	0.876-2.276	0.156
≧̸70y	1.914	1.073-3.413	0.028	1.642		0.918-2.939	0.095	2.071	1.157-3.706	0.014	1.744	0.974-3.124	0.061
**Smoking history** (No/Yes)	1.211	0.722-2.031	0.468										
**Location** (Left/Right)	0.941	0.613-1.443	0.779										
**T stage** (T1/T2a)	3.957	2.602-6.019	<0.001	1.606		1.034-2.493	0.035	1.777	1.151-2.743	0.010	1.755	1.134-2.716	0.012
**Histological pattern**			<0.001				<0.001			<0.001			<0.001
SMPN	Ref			Ref				Ref			Ref		
SMPM	5.019	3.078-8.185	<0.001	2.636		1.582-4.392	<0.001	2.568	1.544-4.271	<0.001	2.504	1.505-4.165	<0.001
SMPP	9.946	5.476-18.064	<0.001	3.781		2.001-7.146	<0.001	3.983	2.116-7.496	<0.001	3.679	1.954-6.930	<0.001
**LVI** (No/Yes)	1.506	0.369-6.137	0.568										
**Radiologic features** (Subsolid/Solid)	3.874	2.346-6.398	<0.001	6.827		2.375-19.625	<0.001	6.845	2.389-19.617	<0.001	6.896	2.410-19.735	<0.001
**NLR** (Low/High)	4.390	2.891-6.666	<0.001	3.756		2.446-5.766	<0.001	NI			NI		
**SII** (Low/High)	3.609	2.358-5.522	<0.001	NI				3.545	2.306-5.449	<0.001	NI		
**SIRI** (Low/High)	2.965	1.957-4.494	<0.001	NI				NI			2.281	1.477-3.524	<0.001

SMPP, solid/micropapillary-predominant; SMPM, solid/micropapillary-minor; SMPN, solid/micropapillary-negative; LVI, lymphovascular invasion; NLR, neutrophil-to-lymphocyte ratio; SII, systemic immune-inflammation index; SIRI, systemic inflammation response index; NI, not included in the multivariate model; Ref, reference, HR, hazard ratio, CI, confidence interval.

**Table 4:** Univariate and multivariate cox regression analysis of factors associated with disease-free survival.

Characteristics	Univariate analysis			Multivariate (model 1 NLR)			Multivariate (model 2 SII)			Multivariate (model 3 SIRI)		
	HR	95%CI	P	HR	95%CI	P	HR	95%CI	P	HR	95%CI	P
**Gender:** Male/Female	0.708	0.514-0.977	0.035	0.960	0.691-1.332	0.805	0.955	0.688-1.325	0.781	1.072	0.764-1.505	0.687
**Age**			0.299									
<60y	Ref											
60-70y	1.019	0.712-1.457	0.918									
≧̸70y	1.391	0.894-2.165	0.144									
**Smoking history** (No/Yes)	1.332	0.901-1.970	0.150									
**Location** (Left/Right)	1.099	0.785-1.540	0.581									
**T stage** (T1/T2a)	6.010	4.296-8.409	<0.001	3.075	2.151-4.396	<0.001	3.198	2.240-4.565	<0.001	3.091	2.164-4.415	<0.001
**Histological pattern**			<0.001			<0.001			<0.001			<0.001
SMPN	Ref			Ref			Ref			Ref		
SMPM	4.620	3.238-6.592	<0.001	2.300	1.581-3.347	<0.001	2.285	1.571-3.322	<0.001	2.280	1.567-3.317	<0.001
SMPP	5.884	3.533-9.800	<0.001	2.230	1.300-3.824	0.004	2.242	1.308-3.844	0.003	2.191	1.276-3.762	0.004
**LVI** (No/Yes)	2.585	1.056-6.328	0.038	1.131	0.457-2.797	0.790	0.946	0.381-2.345	0.904	0.970	0.393-2.398	0.948
**Radiologic features** (Subsolid/Solid)	9.223	4.989-17.051	<0.001	3.965	2.056-7.647	<0.001	3.983	2.068-7.671	<0.001	4.009	2.079-7.730	<0.001
**NLR** (Low/High)	1.909	1.325-2.751	0.001	1.666	1.150-2.413	0.007	NI			NI		
**SII** (Low/High)	1.727	1.182-2.523	0.005	NI			1.679	1.146-2.460	0.008	NI		
**SIRI** (Low/High)	2.059	1.488-2.850	<0.001	NI			NI			1.781	1.267-2.504	0.001

SMPP, solid/micropapillary-predominant; SMPM, solid/micropapillary-minor; SMPN, solid/micropapillary-negative; LVI, lymphovascular invasion; NLR, neutrophil-to-lymphocyte ratio; SII, systemic immune-inflammation index; SIRI, systemic inflammation response index; NI, not included in the multivariate model; Ref, reference; HR, hazard ratio; CI, confidence interval.

### Prognostic Nomogram for CSS and DFS

**Figure 2:**
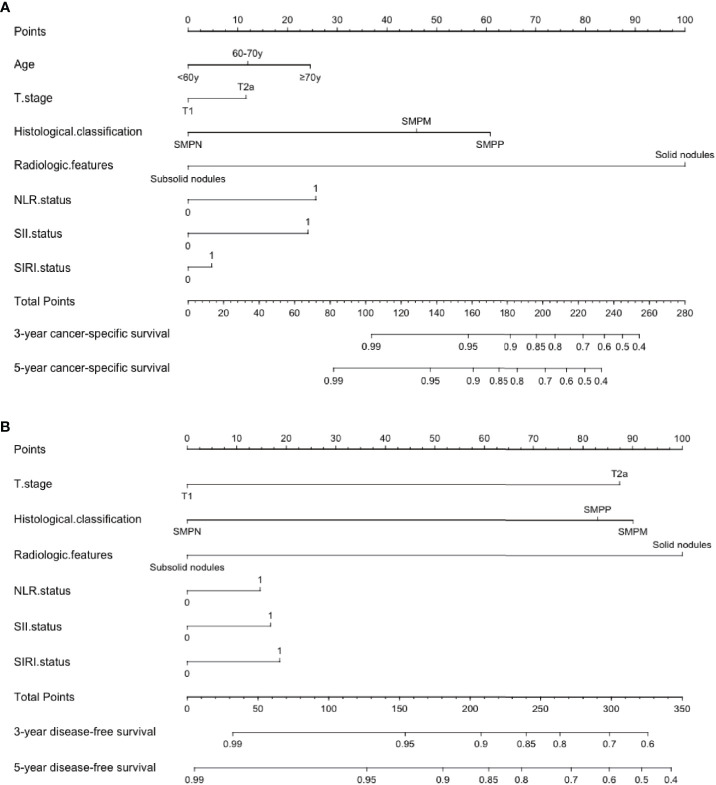
Postoperative prognostic nomogram for predicting **(A)** 3- and 5-year cancer-specific survival probability **(B)** 3- and 5-year disease-free survival probability for patients with stage I adenocarcinoma. SMPP, solid/micropapillary-predominant; SMPM, solid/micropapillary-minor; SMPN, solid/micropapillary-negative; NLR, neutrophil-to-lymphocyte ratio; SII, systemic immune-inflammation index; SIRI, systemic inflammation response index.

Patients were then randomly classified into training cohort and validation cohort in a proportion of 7:3. The clinicopathological characteristics of the two cohorts were shown in **Table S3**. A nomogram was established incorporating the identified significant independent prognostic factors including T stage, histological pattern, radiologic features, NLR, SII and SIRI, which was applied to predict the 3- and 5-year CSS and DFS of the training cohort. Since age showed significant prognosis value in one model (model 2 SII), we included age as an independent factor to construct the nomogram in CSS. The nomogram indicated radiologic features as sharing the largest contribution to predict CSS, followed by histological pattern. NLR, SII, SIRI, age and T stage showed a moderate influence on survival. In the prognosis of DFS, radiologic features, histological pattern and T stage shared relatively the same large contribution, followed by NLR, SII and SIRI status **(****Figure 2****)**. Each factor of these variables in the nomogram was assigned a score on the point scale. By adding up the total score and drawing a vertical line down to locate the score on the total point scale, it was easy to predict the estimated probability of CSS and DFS at each score point.

### Calibration and Validation of the Nomogram

The C-index for the established nomogram to predict CSS was 0.858 (95% CI, 0.840–0.876) in the training cohort and 0.778 (95% CI, 0.738–0.818) in the validation cohort. For DFS, the C-index was 0.830 (95% CI, 0.813–0.847) in the training cohort and 0.758 (95% CI, 0.727–0.789) in the validation cohort, which was superior to the TNM staging system both in the training (CSS: 0.668, 95% CI, 0.636-0.700, DFS: 0.72, 95% CI, 0.696-0.744) and validation set (CSS: 0.652, 95% CI, 0.604-0.700, DFS: 0.695, 95% CI, 0.661-0.729).

**Figure 3:**
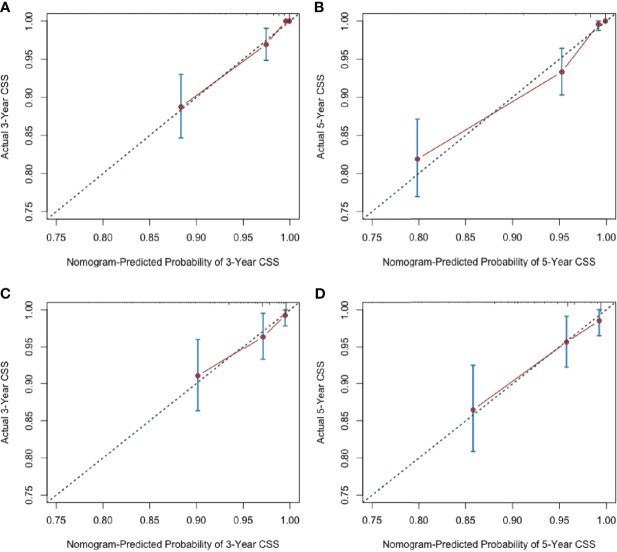
Nomogram calibration curves for predicting cancer-specific survival probabilities **(A)** at 3 year and **(B)** 5 years in the training cohort; at 3 year **(C)** and 5 years **(D)** in the validation cohort.

**Figure 4:**
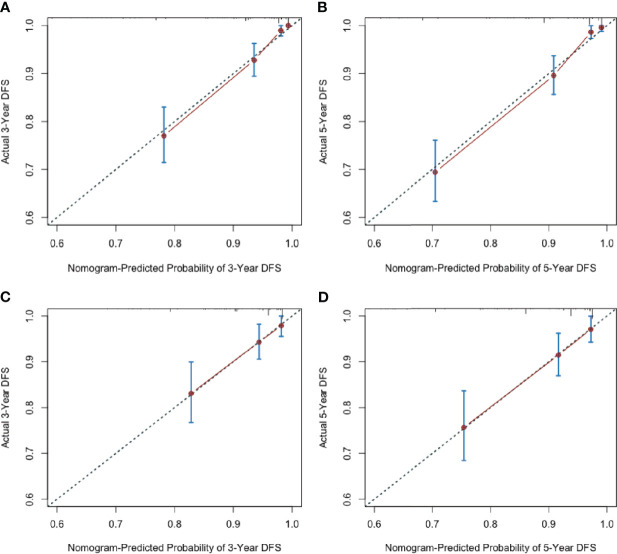
Nomogram calibration curves for predicting disease-free survival probabilities **(A)** at 3 year and **(B)** 5 years in the training cohort; at 3 year **(C)** and 5 years **(D)** in the validation cohort.

Additionally, the calibration plots adjusted by 1000 bootstrapping resamples were performed to verify the performance of the nomogram. The calibration curves of 3-year and 5-year postoperative CSS and DFS probability invalidated a high consistency between the model prediction and the actual observation, both in the training and validation set, demonstrating the nomogram’s reliable repeatability **(****Figures 3**, **4****)**.

**Figure 5:**
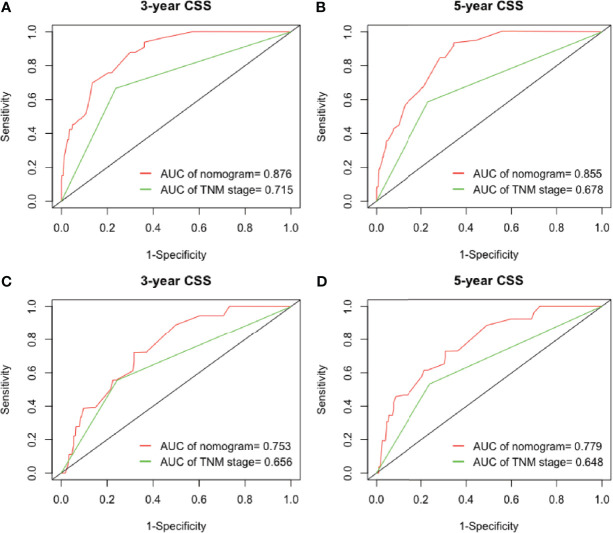
The ability of ROC analysis of nomogram to predict the **(A)** 3-year and **(B)** 5-year CSS rate in the training cohort; **(C)** 3-year and **(D)** 5-year CSS rate in the validation cohort. ROC, receiver operating characteristic; CSS, cancer-specific survival.

**Figure 6:**
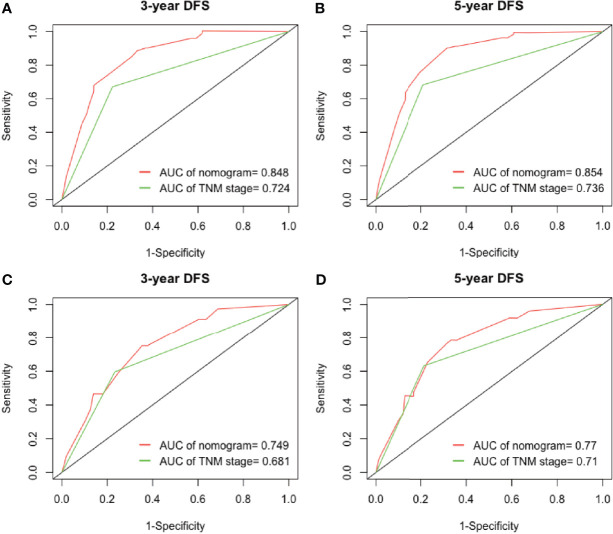
The ability of ROC analysis of nomogram to predict the **(A)** 3-year and **(B)** 5-year DFS rate in the training cohort; **(C)** 3-year and **(D)** 5-year DFS rate in the validation cohort. ROC, receiver operating characteristic; DFS, disease-free survival.

Furthermore, we analyzed the prognostic ability of the nomogram by the ROC analysis, which was then compared with the traditional TNM staging system. In the analysis of the 3- and 5-years CSS, the AUCs of the nomogram were 0.876 and 0.855, respectively, which were both higher than those of the TNM stage (0.715 and 0.678). In the validation cohort, the AUCs of the nomogram were 0.753 and 0.779, which were also higher than those of the TNM stage (0.656 and 0.648) (**Figure 5****)**. In the analysis of the 3- and 5-years DFS, we observed similar results (**Figure 6****)**, which demonstrated that the established nomogram was an accurate measure to predict the postoperative CSS and DFS of stage I adenocarcinoma patients.

### Clinical Significance of the Established Nomogram as the Risk Stratification Indicator

**Figure 7:**
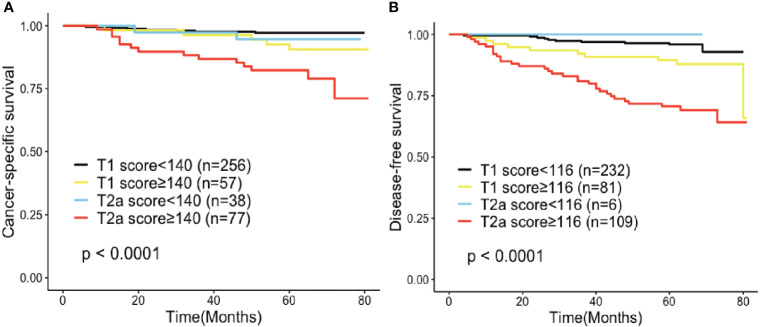
Risk group stratification within each staging system (IA, IB) in **(A)** cancer-specific survival and **(B)** disease-free survival.

Furthermore, the value of the established nomogram on risk stratification in stage I lung adenocarcinoma patients was explored. Using CSS and DFS as endpoints, ROC curves were performed to define the optimal cut-off values of the total score of the established nomogram in the training cohort. Based on the maximum Youden index, the optimal cut-off values were 140 and 116 for CSS and DFS, respectively. After applying them to the validation cohort, we distinguished two risk groups in terms of CSS (low-risk: <140, and high-risk: ≧̸140) and DFS (low-risk: <116, and high-risk: ≧̸116), which successfully stratified patients into high- and low-risk groups within the same T category (**Figure 7****)**.

### Influence of Adjuvant Chemotherapy

Finally, we performed an exploratory research of the predictive value of the established model on adjuvant chemotherapy. We focused on patients with stage IB lung adenocarcinoma. Patients who accepted adjuvant chemotherapy were younger (P<0.001) in comparison with those treated with observation. To reduce selection bias from pretreatment factors, PS matching between the patients who underwent adjuvant chemotherapy (n=180) and those who received observation (n=190) was conducted, and 133 cases in each group were finally matched. All confounding factors before and after PS matching were listed in **Table S4**.

**Figure 8:**
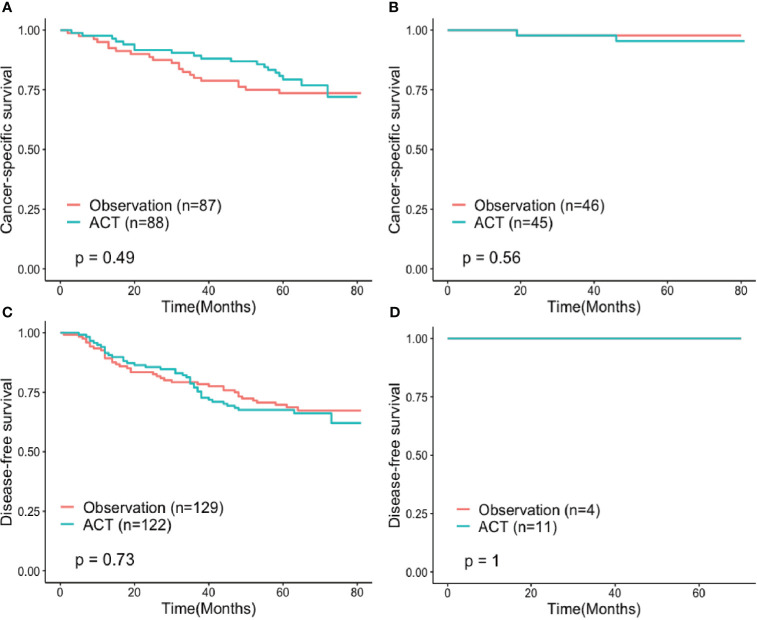
Cancer-specific survival of propensity score-matched **(A)** high-risk and **(B)** low-risk subgroups and disease-free survival of propensity score-matched **(C)** high-risk and **(D)** low-risk subgroups compared between patients treated with observation and adjuvant chemotherapy after surgery. ACT, Adjuvant chemotherapy.

Adjuvant chemotherapy failed to contribute to a survival benefit for both high-risk and low-risk patients, though survival analysis stratified by the score of the established nomogram showed that patients in the high-risk group who received adjuvant chemotherapy seemed to have longer CSS without statistical significance (HR 0.81, 95%CI: 0.432-1.497, P=0.49) (**Figure 8**).

## Discussion

For lung adenocarcinoma, the TNM staging system is widely applied for prognostication. Nevertheless, the clinical prognosis varies even in the same stage. Although the prognosis for stage I lung adenocarcinoma is generally favorable, there is still a probability of locoregional recurrence and distant metastasis (21). A growing body of evidence suggested that inflammation was critical to tumorigenesis, tumor angiogenesis and metastasis (8–10). Tumor-induced inflammatory reactions caused changes in hematology (8). Many studies indicated that systemic inflammatory indices in peripheral blood could help to predict the prognosis of many solid tumors included esophageal squamous cell carcinoma, gastric cancer, gallbladder carcinoma and NSCLC (13, 22–24).

A detailed study measured 51 immune cell compositions in NSCLC, which showed neutrophils were the most prevalent immune cell population (25). Tumor-associated neutrophils (TANs) can be distinguished in N1 and N2 TANs according to their effects on tumor cells. N2 TANs stimulate immunosuppression in the process of chronic inflammatory immune response by DNA instability or releasing cytokines and chemokines, which promote tumorigenesis, tumor growth and metastasis (26, 27). TANs may be a critical source of MMP-9, which can promote vascular endothelial growth factor (VEGF) activation and tumor angiogenesis (28). Recent studies showed that neutrophils could synthesize and secret many inflammatory mediators such as prostaglandin E2, which would enhance cell proliferation in NSCLC (29). Lymphocytes, including T lymphocytes and B lymphocytes, are important parts of the immune system which play a critical role in anti-tumor cell-mediated immunity. The activation of CD8 positive cytotoxic T cells is a key step of the “cancer-immunity cycle” which induces cytotoxic immune response (30). CD8+, CD3+, CD45RO+ memory T cells, and tumor-infiltrating lymphocytes (TILs) are widely confirmed as representative potential powerful prognostic instruments for many solid tumors, including NSCLC. The decline of lymphocytes may result in a worse prognosis (31–35). Myeloid-derived suppressor cells (MDSCs) display immunosuppressive function by inducing immunosuppressive cells, producing reactive oxygen species (ROS) and depleting metabolites which are significant to T cell functions (36). In a meta-analysis, elevated pretreatment circulating MDSCs had a negative impact on prognosis in most solid tumors (37). A study illustrated a phenomenon that there was an accumulation of tumor-associated monocytes in advanced lung tumors compared to nonmalignant distal lung, which was not observed in early-stage tumors (38). Platelets are small cells that circulate in the blood. Tumor cells can activate platelets and stimulate aggregation, which can protect tumor cells from attack of NK cells and shear forces (39, 40). ATP released from tumor-associated platelets into the blood relax endothelial barrier function, which may augment tumor cell extravasation and finally leads to metastasis (41). Platelets can induce tumor cells to release soluble NKG2D ligands to avoid the detection by NK cells and inhibit producing inflammatory cytokine (IFNγ). The platelet-derived TGF-mediated suppression of the CD226/CD96-CD112/CD155 axis further suppresses NK cells (42). Therefore, platelets assist immune evasion. Generally, these various blood counts constitute the specific immune environment that may have a significant impact on tumorigenesis and metastasis.

Several compound systemic inflammatory indices have been elicited to predict the prognosis of tumors. NLR, platelet-lymphocyte ratio (PLR), monocyte-lymphocyte ratio (LMR) and Glasgow Prognostic Score (GPS) were consistently validated to have reliable prognostic values in patients with both operable cancers and advanced inoperable cancers (43, 44). Because of the composition of two inflammatory cells, the prediction ability of these biomarkers for survival outcomes may not be the best. Therefore, more inflammatory indices have been developed recently. SII is calculated based on three parameters: neutrophils, platelets and lymphocytes. SII was first depicted and proved effective as a prognostic predictor in hepatocellular carcinoma (19). A growing number of studies indicated that elevated SII was related to poor prognosis in various malignancies: gallbladder carcinoma (23), breast cancer (45), colorectal cancer (46), renal cell carcinoma (47) and NSCLC (48, 49). SIRI is another biomarker that consists of three types of inflammatory cells, which has also been implied to be a promising prognostic indicator in several cancers: esophageal squamous cell carcinoma (50), gallbladder cancer (51), cervical cancer (20), and NSCLC (24).

Previous studies focused on the role of inflammation biomarkers in NSCLC were summarized in **Table 5**. Most studies included NSCLC patients staged I-IIIA, whose postoperative interventions vary across different stages. As far we know there is insufficient data from researches to focus exclusively on the prognostic value of inflammatory biomarkers in patients with surgically resected stage I lung adenocarcinoma, especially in such a big sample size. Stage I lung adenocarcinoma patients receive less postoperative treatment thus avoiding heterogeneity which may lead to different clinical prognoses. Furthermore, most studies used overall survival (OS) as the endpoint outcome, which ignored death due to other causes rather than lung cancer because of the long survival time of early-stage lung adenocarcinoma. Compared to OS, CSS seems to be more suitable. Last but not least, all studies did not include validation which we examined in our investigation.

**Table 5:** Main characteristics of recent eligible studies (operable-stage NSCLC).

Author Year	Country	Study	Time frame	Sample size	Age	TNM stage	Follow-up	Biomarkers	Cutoff value	Outcome	Validation
Yusuke (52) 2015	Japan	Retro	2000-2008	343	68 (25-87)	I	73.5 (15-159)	NLR	2.5	OS RFS	No
Yuan (53) 2017	China	Retro	2005-2009	1466		I-IIIA	69.9 (64-75)	NLR	2.06	OS	No
								PLR	204		
								MLR	0.35		
Gao (49) 2018	China	Retro	2009-2011	410	60 (35-82)	I-IIIA	54 (3-96)	SII	395.4	OS	No
								NLR	1.9		
								PLR	108.8		
								LMR	3.6		
Huang (18) 2018	China	Retro	2006-2009	589	60 (24-82)	I-IIIA	44	NLR	2.3	OS	No
								Fibrinogen	3.48	DFS	
Guo (48) 2019	China	Retro	2006-2012	569	60 (27-80)	I-III	60.3(0.9-146.7)	SII	419.6	OS	No
								NLR	1.74		
								PLR	88.7		
Li (54) 2019	China	Retro	2013-2015	390		I-IIIA	50 (12-66)	SIRI	0.99	OS	No
								NLR	3.60	DFS	
								PLR	106.13		
								LMR	2.40		
Shoji (55) 2020	Japan	Retro	2006-2012	529	68 (30–91)	IA	63 (0-144)	SII	358	RFS	No
								NLR	1.5		
								PLR	184		
								MLR	0.19		

NLR, neutrophil-to-lymphocyte ratio; SII, systemic immune-inflammation index; SIRI, systemic inflammation response index; MLR, monocyte/lymphocyte ratio; PLR, platelet/lymphocyte ratio; LMR, lymphocyte/monocyte ratio; RFS, recurrence-free survival; OS, overall survival; DFS, disease-free survival.

In this study, we performed ROC curve to calculate the optimal cut-off values of NLR, SII and SIRI which were 2.606, 580.671 and 0.705, respectively, which was similar to previous studies (18, 24). Survival curves indicated that the patients in the high NLR, SII and SIRI groups had a poorer CSS (P < 0.001) and DFS (P < 0.001) than those in the low NLR, SII and SIRI groups, which was consistent with the result of previous studies (24, 48). Because of the strong collinearity, NLR, SII and SIRI were tested separately in multivariate analysis. Our study demonstrated that the significant independent markers for CSS in the multivariate analyses were the same for DFS, which included NLR, SII, SIRI, T stage, histological pattern and the presence of GGO components. Furthermore, we established a nomogram based on these factors. Since all the three inflammatory biomarkers were simple and low-cost, which demonstrated our model’s convenience and economics, we included all variables in the nomogram. The prediction model we established displayed better discriminatory ability compared to the traditional TNM staging system, as shown in the C-index and ROC analysis. The calibration curves displayed good consistency between model prediction and practical observation, which guaranteed repeatability. All these results were subsequently validated by the verification cohort. We then successfully separated patients with distinct prognoses within the same T category by stratifying them into high-risk and low-risk subgroups. Though there was a greater CSS difference for high-risk patients who received adjuvant chemotherapy, our study didn’t suggest that adjuvant chemotherapy improved survival in both high-risk and low-risk stage IB lung adenocarcinoma patients, which proposed a hypothesis that inflammatory biomarkers may act as a prognostic factor, not a predictive factor.

Our study specifically highlighted early-stage surgically resected lung adenocarcinoma patients, which could avoid heterogeneity and reflect the biological behavior of the tumor itself better. We investigated the prognostic value of NLR, SII and SIRI simultaneously in one study and the sample size was relatively large. Furthermore, validation of the model we established was accomplished by bootstrap resampling. Although previous studies have successfully reported nomograms to predict the postoperative survival and recurrence in stage IA NSCLC, they didn’t include inflammatory biomarkers (7, 56–59). To our knowledge, this study is the first large cohort study to create a nomogram combining inflammatory biomarkers and other clinicopathological factors to predict CSS and DFS probability in stage I adenocarcinoma patients. Since all factors in the nomogram were the existing information, it applies to clinical practice easily.

There were several limitations for the present study. Firstly, it was based on retrospective information from a single institution, which may lead to the inevitable selection bias. Secondly, though inflammatory indices were obtained within 1 week before surgery, they may vary over different institutions with different test machines. Therefore, whether the cut-off value applies to other institutions is questionable. Thirdly, many diseases such as autoimmune and rheumatic disease may affect circulating inflammatory cells, which may lead to the instability of the value. Finally, because of the unavailability of the external validation, we could not rule out the bias completely. It is necessary to conduct a multi-institutional, specifically a well-designed prospective randomized controlled trial to confirm our conclusions.

If the clinical significance of the inflammatory biomarkers will be confirmed in the future, the preoperative level of inflammatory indices might not only apply as a parameter for predicting survival outcomes and monitoring treatment response which may help clinicians to precisely implement individualized treatment strategies, but also be considered as a stratification variable in clinical research design for lung adenocarcinoma patients in the future. Adjuvant therapy or anti-inflammatory treatment may contribute to a survival benefit for lung adenocarcinoma patients with a preoperative high inflammatory biomarkers level.

## Conclusions

Generally, this study demonstrated that preoperative NLR, SII and SIRI were simple independent prognostic factors of CSS and DFS in stage I lung adenocarcinoma. Compared to the traditional TNM staging system, the nomogram we established integrating three inflammation-based biomarkers and other clinicopathological indicators can help clinicians identify high-risk stage I lung adenocarcinoma patients more objectively after the operation. Furthermore, we investigated that total points calculated by the nomogram were an appropriate risk indicator that can distinguish CSS and DFS based on the TNM category of stage I lung adenocarcinoma, which will help clinicians provide individualized prognostic consulting. Whether adjuvant chemotherapy could contribute to a survival benefit for high-risk patients is still questionable, which may need further studies.

## Data Availability Statement

The raw data supporting the conclusions of this article will be made available by the authors, without undue reservation.

## Ethics Statement

All procedures in this study adhered to the Declaration of Helsinki (as revised in 2013). The study was approved by the institutional Ethical Committee of Shanghai Chest Hospital (No. KS21002). Since this was a retrospective study, the necessity for written informed consent from patients was waived.

## Author Contributions

Y-JS: Conceptualization, data collection, follow-up, software, and writing original manuscript. L-QQ: Data curation, resources, and editing original manuscript. Z-PD, Q-QL, and HZ: Methodology and resources. W-YX and Y-YF: Data curation. WF, QZ, WY, and X-WC: Conceptualization and methodology. X-LF: Conceptualization, project administration, resources, draft review, and supervision. All authors contributed to the article and approved the submitted version.

## Funding

This work was supported by the Major Research Plan of the National Natural Science Foundation of China (Grant No. 92059206).

## Conflict of Interest

The authors declare that the research was conducted in the absence of any commercial or financial relationships that could be construed as a potential conflict of interest.

The reviewer LC declared a shared affiliation, with no collaboration, with the authors to the handling editor at the time of review.

## Publisher’s Note

All claims expressed in this article are solely those of the authors and do not necessarily represent those of their affiliated organizations, or those of the publisher, the editors and the reviewers. Any product that may be evaluated in this article, or claim that may be made by its manufacturer, is not guaranteed or endorsed by the publisher.
