# Supplementary material for: Prognostic Value of Inflammatory Biomarkers in Patients With Stage I Lung Adenocarcinoma Treated With Surgical Dissection
Source: Front Oncol. 2021 Sep 1;11:711206. doi: 10.3389/fonc.2021.711206 (PMC8440980; doi:10.3389/fonc.2021.711206)
Supplement: Supplementary file 1 [file DataSheet_1.docx]

Supplementary Material

**Supplementary Figure 1** Patient selection

**Supplementary Figure 2** Receiver operating characteristic curve analysis of the optimal cutoff value of the neutrophil-to-lymphocyte ratio (NLR), systemic immune-inflammation index (SII), and systemic inflammation response index (SIRI) in patients with resected stage I lung adenocarcinoma. Areas under the curve for cancer- specific survival were 0.638, 0.627 and 0.659 for NLR, SII and SIRI, respectively (P<0.05)

**Table S1** Univariate and multivariate logistic regression associating clinicopathologic variables with SII

| Characteristics | Univariate analysis | | | Multivariate analysis | | |
| --- | --- | --- | --- | --- | --- | --- |
|  | **OR** | **95%CI** | **P-value** | **OR** | **95%CI** | **P-value** |
| Age, years |  |  | 0.819 |  |  |  |
| <60 | Ref. |  |  |  |  |  |
| 60-70 | 1.101 | 0.816-1.486 | 0.576 |  |  |  |
| ⩾70 | 1.062 | 0.703-1.606 | 0.775 |  |  |  |
| Gender |  |  |  |  |  |  |
| Male | Ref. |  |  |  |  |  |
| Female | 0.835 | 0.633-1.103 | 0.204 |  |  |  |
| Smoking history |  |  |  |  |  |  |
| No | Ref. |  |  |  |  |  |
| Yes | 1.411 | 1.008-1.975 | 0.045* | 1.411 | 1.008-1.975 | 0.045* |
| Location |  |  |  |  |  |  |
| Left | Ref. |  |  |  |  |  |
| Right | 0.939 | 0.706-1.247 | 0.662 |  |  |  |
| T stage |  |  |  |  |  |  |
| T1 | Ref. |  |  |  |  |  |
| T2a | 1.258 | 0.927-1.706 | 0.141 |  |  |  |
| Histological pattern |  |  | 0.285 |  |  |  |
| SMPN | Ref. |  |  |  |  |  |
| SMPM | 1.239 | 0.908-1.690 | 0.177 |  |  |  |
| SMPP | 1.340 | 0.777-2.311 | 0.293 |  |  |  |
| LVI |  |  |  |  |  |  |
| No | Ref. |  |  |  |  |  |
| Yes | 1.447 | 0.529-3.961 | 0.472 |  |  |  |
| Radiologic features |  |  |  |  |  |  |
| Solid | Ref. |  |  |  |  |  |
| Subsolid | 0.980 | 0.739-1.299 | 0.888 |  |  |  |

LVI, lymphovascular invasion; SII, systemic immune-inflammation index; SMPP, solid/micropapillary-predominant; SMPM, solid/micropapillary-minor; SMPN, solid/micropapillary-negative; CI, confidence interval; Ref, reference; OR, odds ratio; *Statistically significant (P < 0.05).

**Table S2** Univariate and multivariate logistic regression associating clinicopathologic variables with SIRI

| Characteristics | Univariate analysis | | | Multivariate analysis | | |
| --- | --- | --- | --- | --- | --- | --- |
|  | **OR** | **95%CI** | **P-value** | **OR** | **95%CI** | **P-value** |
| Age, years |  |  | 0.045* |  |  | 0.090 |
| <60 | Ref. |  |  | Ref |  |  |
| 60-70 | 1.292 | 1.003-1.663 | 0.047* | 1.211 | 0.932-1.574 | 0.152 |
| ⩾70 | 1.451 | 1.035-2.035 | 0.031* | 1.452 | 1.022-2.062 | 0.037* |
| Gender |  |  |  |  |  |  |
| Male | Ref. |  |  |  |  |  |
| Female | 0.323 | 0.254-0.410 | <0.001* | 0.347 | 0.262-0.460 | <0.001* |
| Smoking history |  |  |  |  |  |  |
| No | Ref. |  |  |  |  |  |
| Yes | 2.136 | 1.614-2.826 | <0.001* | 1.076 | 0.774-1.494 | 0.664 |
| Location |  |  |  |  |  |  |
| Left | Ref. |  |  |  |  |  |
| Right | 0.825 | 0.651-1.045 | 0.111 |  |  |  |
| T stage |  |  |  |  |  |  |
| T1 | Ref. |  |  |  |  |  |
| T2a | 1.469 | 1.138-1.895 | 0.003* | 1.262 | 0.957-1.665 | 0.100 |
| Histological pattern |  |  | 0.004* |  |  | 0.442 |
| SMPN | Ref. |  |  |  |  |  |
| SMPM | 1.396 | 1.076-1.811 | 0.012* | 1.177 | 0.889-1.559 | 0.255 |
| SMPP | 1.804 | 1.149-2.834 | 0.010* | 1.226 | 0.758-1.983 | 0.405 |
| LVI |  |  |  |  |  |  |
| No | Ref. |  |  |  |  |  |
| Yes | 0.959 | 0.373-2.469 | 0.931 |  |  |  |
| Radiologic features |  |  |  |  |  |  |
| Solid | Ref. |  |  |  |  |  |
| Subsolid | 0.903 | 0.713-1.143 | 0.395 |  |  |  |

LVI, lymphovascular invasion; SIRI, systemic inflammation response index; SMPP, solid/micropapillary-predominant; SMPM, solid/micropapillary-minor; SMPN, solid/micropapillary-negative; CI, confidence interval; Ref, reference; OR, odds ratio; *Statistically significant (P < 0.05).

**Table S3.** Clinicopathologic characteristics of the training and validation cohort

| Characteristics | Training cohort（n=1003） | | Validation cohort（n=428） | |
| --- | --- | --- | --- | --- |
|  | **N** | **%** | **N** | **%** |
| Gender |  |  |  |  |
| Male | 423 | 42.2 | 184 | 43.0 |
| Female | 580 | 57.8 | 244 | 57.0 |
| Age |  |  |  |  |
| <60y | 448 | 44.7 | 172 | 40.2 |
| 60-70y | 405 | 40.4 | 190 | 44.4 |
| ⩾70y | 150 | 15.0 | 66 | 15.4 |
| Smoking history |  |  |  |  |
| No | 823 | 82.1 | 349 | 81.5 |
| Yes | 180 | 17.9 | 79 | 18.5 |
| Tumor Location |  |  |  |  |
| Left | 363 | 36.2 | 167 | 39.0 |
| Right | 640 | 63.8 | 261 | 61.0 |
| T stage |  |  |  |  |
| T1 | 748 | 74.6 | 313 | 73.1 |
| T2a | 255 | 25.4 | 115 | 26.9 |
| Histological pattern |  |  |  |  |
| SMPN | 675 | 67.3 | 293 | 68.5 |
| SMPM | 265 | 26.4 | 109 | 25.5 |
| SMPP | 63 | 6.3 | 26 | 6.1 |
| Radiologic features |  |  |  |  |
| Subsolid | 416 | 41.5 | 161 | 37.6 |
| Solid | 587 | 58.5 | 267 | 62.4 |
| NLR |  |  |  |  |
| Low | 827 | 82.5 | 349 | 81.5 |
| High | 176 | 17.5 | 79 | 18.5 |
| SII |  |  |  |  |
| Low | 831 | 82.9 | 357 | 83.4 |
| High | 172 | 17.1 | 71 | 16.6 |
| SIRI |  |  |  |  |
| Low | 712 | 71.0 | 317 | 74.1 |
| High | 291 | 29.0 | 111 | 25.9 |

SMPP, solid/micropapillary-predominant; SMPM, solid/micropapillary-minor; SMPN, solid/micropapillary-negative; LVI, lymphovascular invasion; NLR, neutrophil-to-lymphocyte ratio; SII, systemic immune-inflammation index; SIRI, systemic inflammation response index; N, number.

**Table S4** Clinicopathological Characteristics of Patients Before and After PSM

| Characteristics | Before PSM | | | After PSM | | |
| --- | --- | --- | --- | --- | --- | --- |
|  | **Observation**  **(n=190)** | **ACT**  **(n=180)** | **P- value** | **Observation**  **(n=133)** | **ACT**  **(n=133)** | **P- value** |
| Age, years |  |  |  |  |  |  |
| <60 | 58 (30.5%) | 92 (51.5%) | <0.001 | 50 (37.6%) | 54 (40.6%) | 0.128 |
| 60-70 | 84 (44.2%) | 71 (39.4%) |  | 56 (42.1%) | 64 (48.1%) |  |
| ⩾70 | 48 (25.3%) | 17 (9.4%) |  | 27 (20.3%) | 15 (11.3%) |  |
| Gender |  |  |  |  |  |  |
| Male | 94(49.5%) | 90(50%) | 0.919 | 67(50.4%) | 64(48.1%) | 0.713 |
| Female | 96(50.5%) | 90(50%) |  | 66(49.6%) | 69(51.9%) |  |
| Smoking history |  |  |  |  |  |  |
| No | 152(80%) | 143(79.4%) | 0.894 | 108(81.2%) | 107(80.5%) | 0.876 |
| Yes | 38(20%) | 37(20.6%) |  | 25(18.8%) | 26(19.5%) |  |
| Location |  |  |  |  |  |  |
| Left | 68(35.8%) | 66(36.7%) | 0.861 | 48(36.1%) | 46(34.6%) | 0.798 |
| Right | 122(64.2%) | 114(63.3%) |  | 85(63.9%) | 87(66.4%) |  |
| Histological classification |  |  |  |  |  |  |
| Acinar | 88(46.3%) | 66(36.7%) | 0.050 | 60（45.1%） | 53（39.8%） | 0.177 |
| Papillary | 60(31.6%) | 78(43.3%) |  | 42（31.6%） | 58（43.6%） |  |
| Solid | 21(11.1%) | 20(11.1%) |  | 20（15.0%） | 11（8.3%） |  |
| Lepidic | 10(5.3%) | 2(1.1%) |  | 2（1.5%） | 1（0.8%） |  |
| Micropapillary | 3(1.6%) | 3(1.7%) |  | 3（2.3%） | 1（0.8%） |  |
| Others | 8(4.2%) | 11(6.1%) |  | 6（4.5%） | 9（6.8%） |  |
| Radiologic features |  |  |  |  |  |  |
| Subsolid | 22(11.6%) | 17(9.4%) | 0.504 | 5（3.8%） | 11（8.3%） | 0.122 |
| Solid | 168(88.4%) | 163(90.6%) |  | 128（96.2%） | 122（91.7%） |  |
| NLR |  |  |  |  |  |  |
| NLR-low | 147(77.4%) | 137(76.1%) | 0.775 | 102(76.7%) | 104(78.2%) | 0.769 |
| NLR-high | 43(22.6%) | 43(23.9%) |  | 31(23.3%) | 29(21.8%) |  |
| SII |  |  |  |  |  |  |
| SII- low | 153(80.5%) | 145(80.6%) | 0.994 | 107(80.5%) | 109(82.0%) | 0.754 |
| SII-high | 37(19.5%) | 35(19.4%) |  | 26(19.5%) | 24(18.0%) |  |
| SIRI |  |  |  |  |  |  |
| SIRI-low | 129(67.9%) | 115(63.9%) | 0.416 | 84(63.2%) | 89(66.9%) | 0.520 |
| SIRI-high | 61(32.1%) | 65(36.1%) |  | 49(36.8%) | 44(33.1%) |  |

SMPP, solid/micropapillary-predominant; SMPM, solid/micropapillary-minor; SMPN, solid/micropapillary-negative; NLR, neutrophil-to-lymphocyte ratio; SII, systemic immune-inflammation index; SIRI, systemic inflammation response index; ACT, adjuvant chemotherapy
